# 基因融合介导EGFR-TKI获得性耐药

**DOI:** 10.3779/j.issn.1009-3419.2020.101.04

**Published:** 2020-05-20

**Authors:** 宜 邵, 殿胜 钟

**Affiliations:** 300052 天津，天津医科大学总医院肿瘤内科 Department of Medical Oncology, Tianjin Medical University General Hospital, Tianjin 300052, China

**Keywords:** 肺肿瘤, 表皮生长因子受体, 基因融合, 获得性耐药, Lung neoplasms, Epidermal growth factor receptor, Gene fusion, Acquired resistance

## Abstract

具有表皮生长因子受体(epidermal growth factor receptor, *EGFR*)敏感突变的非小细胞肺癌患者对酪氨酸激酶抑制剂(tyrosine kinase inhibitors, TKIs)反应良好，但是最终会发生获得性耐药。近来发现EGFR-TKIs耐药机制除了*EGFR*二次突变、*MET*扩增、组织学转化等外，基因融合的出现也可以介导TKIs耐药。TKIs耐药后可发生转染重排基因(rearranged during transfection, *RET*)，鼠类肉瘤病毒癌基因同源物B1 (v-raf murine sarcoma viral oncogene homolog B1, *BRAF*)，间变性淋巴瘤激酶(anaplastic lymphoma kinase, *ALK*)等多种基因融合，发生率约为1%左右。临床病例及体内体外实验证实基因融合可以介导EGFR-TKI耐药，联合使用EGFR抑制剂和基因融合抑制剂可能是一种有效的治疗方式。对基因融合介导EGFR-TKI耐药的理解有助于后续诊疗策略的制定。

## Abstract (en)

Patients with sensitive epidermal growth factor receptor (*EGFR*) mutations often respond to tyrosine kinase inhibitors (TKIs), but acquired resistance will eventually develop. The most common mechanisms of acquired resistance include secondary *EGFR* mutation, *MET* amplification, and histologic transformation. Besides, gene fusions could also mediate the process of acquired resistance. Various gene fusions including rearranged during transfection (RET), v-raf murine sarcoma viral oncogene homolog B1 (BRAF) and anaplastic lymphoma kinase (ALK) could take place after TKIs resistance, the incidence of which is around 1%. The clinical cases and experiments both in vitro and in vivo have proved the role of gene fusions in EGFR-TKI resistance. The combination of EGFR inhibitors and gene fusion inhibitors might be an effective therapeutic method. The understanding of gene fusions at EGFR-TKI resistance may contribute to the subsequent diagnosis and treatment strategy.

肺癌是全球范围内发病率和死亡率最高的肿瘤，其中非小细胞肺癌(non-small cell lung cancer, NSCLC)约占肺癌的80%^[1]^。具有表皮生长因子受体(epidermal growth factor receptor, *EGFR*)敏感突变的患者对酪氨酸激酶抑制剂(tyrosine kinase inhibitors, TKIs)具有良好反应，一代TKIs的总反应率为55%-80%，无进展生存期(progression-free survival, PFS)为9个月-14个月^[2, 3]^。一代TKIs最常见的耐药机制是EGFR T790M突变，三代TKI药物奥希替尼对此类突变具有良好疗效^[4]^。随着耐药后再活检的广泛应用，人们对于耐药机制的理解逐渐深入，EGFR-TKIs耐药机制除了*EGFR*突变(一代药物的T790M突变，三代药物的C797S突变)，肝细胞生长因子受体*MET*扩增，组织学转化等外^[5, 6]^，近来发现基因融合的出现也是一种少见但确切的耐药机制。本文将EGFR-TKIs耐药后各种基因融合现象综述如下。

## 1 耐药融合的发现和发生率

2015年，Klempner等^[7]^报道2例具有*EGFR* del19突变的肺癌患者，分别使用厄洛替尼9个月和10个月后耐药，对于耐药前后的组织标本进行全面基因组测序(comprehensive genomic profiling, CGP)，发现耐药后标本同时存在*EGFR* del19突变和卷曲螺旋结构域蛋白6 (coiled coil domain containing 6, CCDC6) -转染重排(rearranged during transfection, *RET*)原癌基因融合，而没有*EGFR* T790M突变、*MET*扩增等其他耐药机制。使用EGFR-TKI之前的标本没有RET融合。这是最早关于基因融合可能介导EGFR-TKI耐药的报道。

Piotrowska等^[8]^使用锚定多重聚合酶链式反应(polymerase chain reaction, PCR)方法回顾性分析起初具有*EGFR*突变、使用厄洛替尼，吉非替尼或阿法替尼耐药患者的肿瘤组织，发现1例阿法替尼耐药患者具有*CCDC6-RET*融合，1例化疗/奥希替尼进展后鼠类肉瘤病毒癌基因同源物B1 (v-raf murine sarcoma viral oncogene homolog B1, *BRAF*)融合，还有1例阿法替尼/西妥昔单抗进展后二代测序(next generation sequencing, NGS)检测到核受体共激活因子4 (nuclear receptor coactivator 4, *NCOA4*) -*RET*融合。

对32例使用三代EGFR-TKI奥希替尼耐药后患者的35份组织标本、26份循环肿瘤DNA (circulating tumor DNA, ctDNA)进行检测，1例患者血浆中发现CCDC6-RET和原肌球蛋白3 (tropomyosin 3, TPM3) -神经营养性酪氨酸受体激酶1 (neurotrophic receptor tyrosine kinase 1 gene, *NTRK1*)融合。锚定多重PCR方法对24例具有足够组织标本的患者进行检测，发现1例*CCDC6-RET*融合，1例*PCBP2-BRAF*融合，1例甘油酯激酶(acylglycerol kinase, *AGK*) -BRAF融合。值得注意的是，这3例同时都伴有T790M突变的丢失，这可能表明了新出现的融合代表了旁路机制的活化^[8]^。

分析3, 505例具有*EGFR*突变或接受过EGFR-TKIs治疗的NSCLC患者肿瘤或血液标本，发现31例(0.88%)同时具有融合：包括10例(32%) BRAF，7例(23%)间变性淋巴瘤激酶(anaplastic lymphoma kinase, ALK)，6例(19%) RET，6例(19%)成纤维细胞生长因子受体3 (fibroblast growth factor receptor 1, FGFR3)，1例(3.2%) EGFR，1例(3.2%) NTRK1。其中12例患者具有治疗前后配对标本，在TKI治疗前不存在融合。3例使用奥希替尼耐药者，融合出现的同时伴有T790M丢失，且具有获得性融合的肿瘤，肿瘤突变负荷(tumor mutational burden, TMB)水平较低(中位，3.5突变/Mb)^[9]^。

3, 014例具有*EGFR*突变的NSCLC患者进行组织或ctDNA基因组测序，发现28例(0.9%)同时具有活化融合(*BRAF* 12例，*FGFR3* 5例，*RET* 5例，*ALK* 4例，*NTRK1* 1例，*EGFR* 1例)，其中25例没有合并其他耐药机制，而21例可追溯病史的患者都使用过EGFR-TKI治疗。10例具有治疗前后配对标本发现TKI治疗前不存在融合，因而确定为获得性融合[FGFR3-转化酸性含卷曲螺旋蛋白3 (transforming, acidic coiled-coil containing protein 3, TACC3) 4例，棘皮动物微管相关蛋白样4 (echinoderm microtubule-associated protein-4, EML4)-ALK 2例，CCDC6-RET 2例，AGK-BRAF 1例，TPM3-NTRK1 1例]，其中3例(2例FGFR3和1例BRAF)配对的融合伴有T790M丢失^[10]^。

综上，EGFR-TKI耐药后可出现*RET*、*BRAF*、*ALK*等多种基因融合。基因融合在EGFR-TKI获得性耐药机制中发生率不足1%，属于介导获得性耐药的少见事件。现将各种基因融合分述如下。

## 2 *RET*融合

### 2.1 原发性*RET*融合

*RET*基因是一种位于10号染色体长臂上的原癌基因(10q11.2)，其编码的RET蛋白是一种酪氨酸激酶受体，结合配体后刺激胞内区域发生磷酸化，激活下游信号，进一步参与调节细胞的生长和分化^[11]^。*RET*基因自身断裂后与其他基因接合发生重组，成为一个新的融合基因，使RET酪氨酸激酶的活化脱离配体的调控，发生自我磷酸化，从而促使原癌基因的转化，引发肿瘤生成^[12]^。2011年，1例肺腺癌中发现驱动蛋白家族成员5B (kinesin family member 5B, *KIF5B*) -*RET*融合，由*KIF5B*基因的第16号外显子末端与*RET*的12外显子起始端融合而成，被认为是部分肺癌的驱动基因^[13]^。

*RET*融合在肺癌患者中阳性率为1%-2%^[12]^，并常与*EGFR*、*KRAS*、*ALK*、*BRAF*等其他基因改变相排斥^[13, 14]^。Takeuchi等^[15]^对1, 114例肺腺癌患者进行检测，共发现14例(1.2%)患者具有*RET*融合，这些患者女性多见，不吸烟或轻吸烟，且EGFR和KRAS阴性。与RET基因发生融合突变的基因包括KIF5B^[13]^、CCDC6^[16]^、三基序蛋白33 (tripartite motif containing 33, TRIM33)^[17]^、NCOA4^[18]^，其中在NSCLC中KIF5B-RET型最常见^[18]^。

### 2.2 EGFR-TKI获得性耐药后*RET*融合

对EGFR-TKIs发生获得性耐药后，*RET*融合的发生率为0.2%-4.2%^[8-10]^。但是基因融合出现的确切机制还不清楚。由于未见治疗前*EGFR*突变和*RET*融合共存的报道，因此有可能在长期EGFR抑制的诱导下肺癌细胞出现了旁路机制的活化，但是也不能完全除外治疗前即存在*RET*融合的小克隆，在EGFR抑制后此部分克隆逐渐变成优势的可能。

Rich等^[19]^分析176例使用EGFR-TKIs后出现RET改变患者中，发现del19比L858R突变患者的*RET*融合发生率高(0.8% *vs* 0.2%, *P* =0.04)，在伴有T790M和/或C797S的患者中比没有这两个突变的患者发生率更高(1.1% *vs* 4.6% *vs* 0.6%)。RET融合在奥希替尼耐药后发生率(9/184, 4.9%)高于一二代TKI (13/1627, 0.8%, *P* =0.000, 1)。Offin等^[20]^同样发现三代TKI后出现*RET*融合的频率更高。174例厄洛替尼或阿法替尼后未检测到融合，而14例奥希替尼后检出3例。但还不清楚是奥希替尼富集获得性融合的潜力更强，还是多线EGFR抑制后反复筛选的结果。

Piotrowska等^[8]^检测41例奥希替尼耐药患者，发现2例获得性*CCDC6-RET*融合。在PC9和MGH134细胞(含有*EGFR* L858R/T790M突变)中表达CCDC6-RET后，细胞对阿法替尼和奥希替尼等EGFR-TKIs耐药，选择性RET抑制剂BLU-667和卡博替尼则可使之敏感性恢复。2例*EGFR*突变阳性患者使用阿法替尼和奥希替尼后耐药，分别获得*CCDC6-RET*和*NCOA4-RET*融合，接受奥希替尼+BLU-667治疗，耐受性良好且迅速反应。研究在细胞和临床层面证实*RET*融合介导EGFR抑制剂耐药，且这个旁路可被选择性RET抑制剂有效抑制。另有研究^[20]^在PC9细胞中表达*CCDC6-RET*和*KIF5B-RET*融合，RET融合可以导致具有*EGFR*突变细胞系奥希替尼耐药，奥希替尼降低母代细胞的EGFR和ERK1/2，而不降低子代细胞的ERK1/2磷酸化。联合卡博替尼后对奥希替尼反应恢复。*RET*融合不改变奥希替尼抑制EGFR磷酸化，证实确实是旁路机制导致耐药。

3, 505例*EGFR*突变患者组织活检，发现6例*RET*融合，包括1例阿法替尼耐药后出现L858R和*NCOA4-RET*融合患者，使用卡博替尼+阿法替尼稳定7个月，与前文所述细胞实验结果一致^[9]^。综上，对于EGFR-TKI耐药后出现*RET*融合的患者，继续EGFR-TKI联合RET抑制剂可能是一种合理的治疗选择。

## 3 *BRAF*融合

### 3.1 原发性*BRAF*融合

*BRAF*基因是1988年由Ikawa等^[21]^首先在人类尤文氏肉瘤中发现的，该基因位于染色体7q34，编码丝氨酸/苏氨酸蛋白激酶，在恶性肿瘤形成、发展过程中发挥重要作用。BRAF蛋白在丝裂原活化蛋白激酶(mitogen-activated protein kinase, MAPK)/细胞外调节蛋白激酶(extracellular regulated protein kinases, ERK)信号通路中起着关键作用，进而调节细胞内的生物学过程^[22]^。2005年，首先在甲状腺癌中报道A型激酶锚定蛋白9 (A-kinase anchoring protein9, *AKAP9*) - *BRAF*融合为一种激活MAPK信号通路的新机制^[23]^。而NSCLC中*BRAF*融合的发生率为4.3%^[24]^。2017年美国临床肿瘤学会上，有报道通过NGS在17, 128例NSCLC患者中发现42例(0.25%) *BRAF*融合现象，其中32例(76.19%)为腺癌，融合伴侣以AGK最为常见，占7.14%，其他还包括TRIM24、细胞质分裂付出蛋白4 (dedicator of cytokinesis 4, DOCK4)和犰狳重复含蛋白10 (armadillo repeat containing 10, ARMC10)等^[25]^。

未经治疗的肺癌患者同时存在*BRAF*融合和*EGFR*突变的报道不多，仅在AURA研究中报道1例使用奥希替尼前NGS发现*BRAF*融合^[26]^。

### 3.2 EGFR-TKI获得性耐药后*BRAF*融合

Yu等^[27]^检测136例经过TKI治疗的具有*EGFR*突变患者，发现2例*BRAF*融合。对比治疗前后标本，BRAF改变分别为1%和5.1%。但研究也只能证实TKI富集了*BRAF*改变，不能证实融合为获得性。

Vojnic等^[28]^检测374例转移性*EGFR*突变肺癌患者，其中174例为TKI治疗后，其中38例有配对TKI前样本。研究共发现4例患者(2例厄洛替尼后，2例厄洛替尼序贯奥希替尼后)具有*BRAF*融合[3例AGK-BRAF，1例泛素蛋白连接酶Praja-1 (praja ring finger ubiquitin ligase 2 gene, PJA2)-BRAF]。4例患者中2例有TKI前标本，都是*BRAF*融合阴性。而在200例TKI前的标本中没有检出同时性*BRAF*融合。

基因编辑将*BRAF*融合基因导入*EGFR*突变细胞系(H1975, HCC827, PC9)以及19del+PJA2-BRAF的原代细胞MSK-LX138cl后，细胞对奥希替尼耐药，BRAF、丝裂原活化蛋白激酶激酶(mitogen-activated protein kinase kinase, MEK) 1/2、ERK1/2和信号传导及转录激活蛋白(signal transducer and activator of transcription 3, STAT3)的磷酸化增加，奥希替尼可以阻断母代细胞而不阻断子代细胞MEK1/2、ERK1/2和STAT3的磷酸化，BRAF敲除后奥希替尼敏感性恢复。MEK抑制剂曲美替尼和奥希替尼可以协同抑制细胞生长，泛RAF抑制剂单药也可抑制具有突变*EGFR*和*BRAF*融合细胞系的生长，因而从细胞水平证实了*BRAF*融合是EGFR-TKI获得性耐药的机制，联合EGFR和MEK抑制或BRAF抑制可能可以克服耐药^[28]^。因此，对于此类患者，联合抑制MEK和EGFR以及抑制*BRAF*融合可能是合适的治疗方案。

## 4 *ALK*融合

### 4.1 原发性*ALK*融合

*ALK*基因位于2p23.2，编码属于胰岛素受体超家族的Ⅰ型跨膜酪氨酸激酶蛋白。2007年，1例27岁肺癌患者中发现*ALK-EML4*基因重排，患者2号染色体短臂中存在倒位，使*EML4* 基因和*ALK*基因的外显子连接，从而形成融合基因*ALK-EML4*^[29]^。重排后融合基因编码的嵌合蛋白含有ALK的酪氨酸激酶结构域，结构域的异常表达使得ALK下游信号通路异常活化而具有致癌性。*ALK*基因重排在NSCLC的总体发生率约为4%^[30]^。肺癌患者中还存在其他融合伴侣，但以*EML4-ALK*最为常见^[31]^。具有*ALK*融合的肺癌患者，使用克唑替尼、阿来替尼等ALK-TKI治疗有效^[32]^。

Yang等^[33]^对977例中国NSCLC患者进行基因检测，发现13例(1.3%)同时存在*EGFR*突变和*ALK*融合。Lee等^[34]^对444例韩国肺腺癌患者进行检测，4例(0.9%)患者同时存在*EGFR*突变和*ALK*融合。Dana-Farber癌症研究院检测了50例NSCLC患者，发现3例(6%)同时具有*EGFR*突变和*ALK*融合^[35]^。因此，存在原发性*EGFR*突变与*ALK*融合并存的现象，但非常少见。发生双重基因改变的患者多使用一代EGFR-TKI或ALK-TKI单药治疗，各个病例报道反应不一，有效率约为60%^[36]^。

### 4.2 EGFR-TKI获得性耐药后*ALK*融合

2016年，Liang^[37]^报道1例*EGFR* del19突变NSCLC患者，检测EML4-ALK阴性。先后使用厄洛替尼HY-15772两种EGFR-TKI治疗8个月后疾病进展。行ctDNA检测，血浆发现*EGFR*突变和*EML4-ALK*重排并存，对ALK抑制剂治疗有反应。

对3, 505例使用EGFR-TKI治疗的患者进行检测，发现7例(0.20%) *ALK*融合^[9]^。新发*ALK*的融合伴侣包括EML4 (*n* =4)、钙调素结合蛋白(striatin, STRN)(*n* =1)、*TRK*融合基因(TRK-fused gene, *TFG*)(*n* =1)、和含普列克底物同源物域家族A成员7 (pleckstrin homology domain containing A7, PLEKHA7)(*n* =1)。出现*STRN-ALK*融合的患者同时伴有T790M丢失，对单药克唑替尼无反应。而奥希替尼耐药后出现新的*PLEKHA7-ALK*融合患者对奥希替尼联合阿来替尼的治疗有反应。因此，对于EGFR-TKI耐药后出现*ALK*融合的患者，继续EGFR-TKI联合ALK-TKI可能是一种较好的治疗选择。

## 5 *FGFR3*融合

*FGFR3-TACC3* 融合是膀胱癌等多种肿瘤的常见驱动基因^[38]^，肺腺癌、肺鳞癌和未分类NSCLC中也都报道过。576例肺腺癌患者行NGS检测，发现*FGFR3-TACC3*融合的总发生率为0.5%。*FGFR3-TACC3*融合导致Ba/F3细胞白介素-3非依赖性生长，细胞对泛FGFR和选择性FGFR抑制剂敏感，但是对EGFR抑制剂吉非替尼耐药^[39]^。

FGFR3-TACC3可能介导EGFR-TKI的耐药。一项研究比较136例EGFR-TKI治疗患者耐药前后的标本，发现FGFR3改变在治疗后更为常见(0.5% *vs* 3.7%, *P* =0.042)^[27]^。对17, 319例肺癌组织(141, 701例腺癌，3, 149例非特指型)行CGP分析，发现5例EGFR-TKI耐药后存在*FGFR3-TACC3*融合并伴有原活化*EGFR*突变，包括1例使用厄洛替尼后，1例使用阿法替尼后，1例使用奥希替尼后和1例使用三代药物ASP8273后^[40]^。

细胞和动物实验证实，*FGFR3-TACC3*融合可以在头颈部鳞癌移植物模型中活化ERK信号通路，逃避EGFR/红白血病病毒癌基因同源物3 (erythroblastic Leukemia Viral Oncogene Homolog 2, ERBB3)阻滞。联合使用阻滞EGFR和ERBB3的抗体时，EGFR阻滞优先抑制ERK活化，而ERBB3阻滞抑制蛋白激酶B (protein kinase B, PKB/AKT)活化。此外，肺癌细胞系NCI-H1975中(*EGFR* L858R+T790M突变)，引入FGFR3-TACC3可导致奥希替尼耐药，而不导致磷脂酰肌醇3-激酶(phosphatidylinositol 3'-kinase, *PI3K*)突变细胞系的PI3K抑制剂耐药^[41]^。但是现在还没有获批的抑制FGFR3融合的药物，因此出现*FGFR3-TACC3*融合后的最佳治疗仍在探索中。

## 6 *NTRK1*融合

之前研究报道结直肠癌和甲状腺癌中出现NTRK1和肌凝蛋白磷酸酶Rho相互作用蛋白(myosin phosphatase Rho-interacting protein, MPRIP)、CD74、TPM3或TFG的融合^[42]^。融合导致结构性TRKA激酶活性增加，可作为癌基因介导肿瘤产生。在3种常用于评估致癌性的非癌症细胞系293T细胞、NIH3T3纤维母细胞和Ba/F3细胞中表达MPRIP-NTRK1和CD74-NTRK1的cDNA，发现表达嵌合蛋白和TRKA自身磷酸化，肿瘤发生锚定非依赖性生长，并导致裸鼠成瘤。而在非肿瘤细胞或对照样本中不存在此种融合。3/91例(3.3%)没有已知癌基因改变的肺癌患者使用NGS或荧光原位杂交检测存在*NTRK1*基因融合^[43]^。一些靶向药物，如拉罗替尼、恩曲替尼和Loxo-195等已经获得FDA批准或试验证实对NTRK1融合有效。

3, 505例具有*EGFR*突变的NSCLC患者，发现1例(0.03%)同时具有*NTRK1*融合，但不确定融合是使用TKI之前还是耐药后出现^[9]^。EGFR-TKI耐药后也可出现*NTRK*融合。32例TKI耐药患者，1例血检发现同时具有*CCDC6-RET*和*TPM3-NTRK1*融合^[8]^。由于报道例数较少，NTRK1介导EGFR的具体机制还未证实，TKI耐药后发生*NTRK1* 融合的最佳治疗也未可知。

## 7 结论

之前未发现融合是介导EGFR-TKI耐药的机制，可能是由于当时使用的基因检测平台未包括融合的检测，而易位断裂点经常发生在内含子区域，聚焦的NGS只检测外显子，因而可能错过这些异常。因此，推荐EGFR-TKI耐药后再活检和使用能够发现激酶融合的基因组测序平台。

一代TKI和三代TKI获得性耐药后基因融合的检出率似有不同，三代药物耐药后融合的发生率高于一代药物(二者分别是3.1%-12.5%和不足1%)^[8, 9, 19, 20]^，但是不能确定是由于三代药物对于基因融合的诱导能力更强，还是EGFR-TKIs序贯使用反复筛选的结果。大部分时候，T790M突变丢失和替代途径耐药机制相关，因此三代TKI耐药后出现T790M丢失的患者应格外注意融合的检测。另外发生获得性融合后TMB水平较低，因此免疫治疗不一定具有良好效果，而无论对于一代还是三代药物来说，联合使用EGFR-TKI和融合抑制剂可能是合理的治疗选择，但是应注意联合用药的毒性。

总之，获得性激酶融合是EGFR-TKIs罕见但是肯定的获得性耐药机制。在EGFR抑制过程中，需要在进展时使用能够检测包括融合在内的各种基因变化，以明确耐药机制，提供治疗决策。
